# Clinical profile of COVID-19 among vaccinated and non-vaccinated patients in India

**DOI:** 10.6026/973206300212872

**Published:** 2025-08-31

**Authors:** Aashna Saith, Gaurav D., Arathi Darshan

**Affiliations:** 1 Jawaharlal Nehru Medical College, Belgaum, Karnataka, India; 2 Department of Medicine, Jawaharlal Nehru Medical College, Belgaum, Karnataka, India

**Keywords:** COVID-19, vaccinated, non-vaccinated, comorbidities, mortality

## Abstract

The clinical profile and disease progression among 101 hospitalized COVID-19 patients, comparing 75 vaccinated and 26 non-vaccinated individuals is of interest. Inflammatory marker IL-6 was significantly higher in the non-vaccinated group (P<0.05), while LFT and RFT showed no notable differences. Non-vaccinated patients had lower oxygen saturation at admission and higher ICU admissions and CT scores. Comorbidities like diabetes and hypertension were linked to increased mortality. Thus, vaccinated patients demonstrated better clinical outcomes and fewer complications.

## Background:

A cluster of severe atypical respiratory disease cases was documented in Wuhan, China, in December 2019. This rapidly spread from Wuhan to other regions of China. The novel coronavirus was designated severe acute respiratory syndrome coronavirus-2 (SARS-CoV-2, 2019-nCoV) due to its significant similarity (80%) with SARS-CoV, which resulted in acute respiratory distress syndrome (ARDS) and elevated fatality rates 2002-2003 [1]. During the pandemic, several notable mutations have emerged, including Alpha (B.1.1.7), Beta (B.1.351), Gamma (P.1), Delta (B.1.617.2) and Omicron (B.1.1.529). These variants exhibit alterations in the Receptor Binding Domain (RBD) and N-Terminal Domain (NTD), except for the Delta variant, which increases the spike protein's affinity for Angiotensin Converting Enzyme 2 (ACE 2) receptors, thereby enhancing viral attachment and subsequent cellular entry. RBD, along with NTD, is the predominant neutralizing target, facilitating the generation of antibodies in response to antisera or vaccines [2]. The SARS-CoV-2 virus predominantly impacts the respiratory system, exhibiting a wide spectrum of symptoms from mild manifestations to severe hypoxia accompanied by acute respiratory distress syndrome (ARDS). The interval between the commencement of symptoms and the onset of ARDS was as little as 9 days in the aforementioned Wuhan report, suggesting that respiratory symptoms may escalate swiftly [3]. Symptoms of lower respiratory tract infection, including fever, dry cough and dyspnea, were documented. Additionally, patients exhibited headaches, dizziness, widespread weakness, vomiting and diarrhea. Individuals with comorbidities face an elevated risk of mortality [4]. Diabetes mellitus exhibited a heightened frequency of death and morbidity, demonstrating a strong correlation with disease development in COVID-19 infection [5, 6]. COVID-19 infection manifests with various hematological abnormalities, including leucopenia and lymphocytopenia, which are indicative of patient prognosis, as well as thrombocytopenia, which correlates with illness severity; paradoxically, an elevated platelet count is linked to a worse outcome. Abnormal liver function tests became more prominent within two weeks of admission, correlating with increased illness severity [7, 8]. Multiple inflammatory indicators, including Procalcitonin (PCT), Serum Ferritin, Erythrocyte Sedimentation Rate (ESR), C - reactive protein (CRP) and IL-6, have been linked to a heightened risk of severe COVID-19 infection. Sepsis is one of the most prevalent consequences observed, followed by respiratory failure, acute respiratory distress syndrome (ARDS), heart failure, and septic shock. Ventilator-associated pneumonia and subsequent infections account for mortality in fifty percent of non-surviving patients [9]. Eight vaccines have been identified in India: Covishield by the Serum Institute of India, Covaxin by Bharat Biotech Ltd, ZyCoV-D by Cadila Healthcare (ZydusCadila), an unnamed COVID vaccine by Biological E. Limited, Sputnik V by Dr. Reddy's Laboratories and an unnamed mRNA vaccine by Gennova Biopharmaceuticals Ltd. The most prevalent vaccines in India are Covishield and Covaxin [10]. Therefore, it is of interest to evaluate the clinical profile of COVID-19 in vaccinated and non-vaccinated patients at a tertiary care hospital, Belagavi, India.

## Materials and Methods:

This is a retrospective cross-sectional research involving 101 COVID-19 patients during the third wave, which occurred from December 2021 to February 2022. The data of COVID-positive patients was acquired from the Medical Records Department at KLE's Dr. Prabhakar Kore Hospital and Medical Research Centre following requisite approvals. Ethical clearance granted by the JNMC Institutional Ethics Committee, letter number MDC/JNMCIEC/417, dated 11/07/2022, to Dr. Aashna Saith and Dr. Gaurav D, for the study entitled "To Study the Clinical Profile of COVID-19 Disease in Vaccinated and Non-Vaccinated Patients at a Tertiary Care Hospital, Belagavi." Individuals aged over 18 years, with positive results from a Rapid Antigen Test (RAT), Reverse Transcription Polymerase Chain Reaction (RT-PCR), or Cartridge-Based Nucleic Acid Amplification Test (CBNAAT) for COVID-19, and without a prior history of COVID-19 infection, which were hospitalized at KLE Hospital, were included in the study. Patients under 18 years having a prior history of COVID-19 infection and negative results from RAT, RTPCR, or CBNAAT were excluded from the research. The study variables encompassed socio-demographic information, a comprehensive medical history including presenting complaints and vaccination status, comorbidities such as diabetes mellitus and hypertension, the CT severity index, laboratory investigations including complete blood count, renal function tests, liver function tests, and a COVID profile comprising D-dimer, serum ferritin, lactate dehydrogenase (LDH), interleukin-6 (IL-6), high-sensitivity C-reactive protein (HsCRP), vital signs such as pulse rate, blood pressure, respiratory rate, random blood sugar, and oxygen saturation (SpO2) levels upon admission, history of ICU admission, mode of oxygenation, and administered treatments.

## Statistical analysis:

The data was transcribed from hard copies into an Excel spreadsheet, and random verification of patient data was conducted to ensure accuracy throughout data entry. The analysis was conducted utilizing SPSS version 20.0. IBM Corp. Released in 2020.IBM SPSS Statistics for Windows, Version 27.0. Armonk, New York: IBM Corporation. Descriptive statistics for quantitative variables were expressed as mean ± standard deviation. The unpaired t-test or Mann-Whitney U-test was employed to compare continuous variables. A p-value of less than 0.05 was deemed statistically significant.

## Results:

A total of 101 COVID-positive patients were admitted to a tertiary care hospital from 1st December 2021 to 28th February 2022, of which 75 patients had received the vaccine against SARS-CoV-2, CoV 2 while 26 patients remained non-vaccinated. 64 patients in the vaccinated group received Serum Institute of India's Covishield, while 11 patients received Bharat Biotech Ltd'sCovaxin. Of the vaccinated group, 9 received a single dose of the vaccine, and the remaining 65 received 2 doses. Fever, cough, dyspnea, vomiting and altered sensorium were some of the most common complains that the patients presented with at the time of admission, it was observed that most of the vaccinated patients developed fever (60%) and cough (48%) whereas most of the non-vaccinated patients presented with dyspnea (69.2%), vomiting (19.2%) and altered sensorium (15.4%) (Figure 1 - see PDF). The SpO2 measured at the time of admission averaged 84.92% and 91.53% in non-vaccinated and Vaccinated patients, respectively (p=0.008), while the average CT score was higher in the non-vaccinated (10.5) than the vaccinated patients (8.79) but was not significant (Table 1 - see PDF). It was noted that a majority of the vaccinated individuals (42.66%) did not require oxygen support. Between the two groups, it was observed that 19.23% and 15.38% of the non-vaccinated group required Continuous Positive Airway Pressure (CPAP) and ventilator support, respectively, in contrast to 8% and 12% of the vaccinated group. Furthermore, COVID markers such as IL6 averaged 851.7 in non-vaccinated individuals in comparison to 161.0 in the vaccinated group (p <0.05); similarly, there was a difference in D-Dimer, HSCRP, Serum Ferritin, and Serum LDH, but it was statistically insignificant (Table 2 - see PDF). Also, patients who received Covaxin showed lower marker values compared to those who received Covishield, although it was statistically insignificant. In our study, we noted that 41.6% of the patients were hypertensive, 37.6% were diabetic, and an unexpected 34.7% did not have any comorbidity. Figure 2 (see PDF) shows the effect of DM and HTN on the outcome of the disease, wherein most of the patients who succumbed to the disease were diabetic or hypertensive. As shown in the figures, diabetics and hypertensive patients are more likely to be symptomatic (Figures 3, 4 - see PDF). In our study, we also observed that among the vaccinated patients, the various COVID markers were higher in diabetics than non-diabetics, where the only significant finding was in HSCRP (p=0.009) and serum LDH (p=0.005) (Table 3, 4 - see PDF).

## Discussion:

Vaccination improved illness progression in our study. Atypical symptoms such as dyspnea, vomiting, and altered sensorium were seen more in non-vaccinated patients 34.6%, 69.2%, and 19.2% compared to vaccinated patients 37.3%, 6.7%, and 4%, possibly due to the vaccine's increased immune response. Fever and cough were the most prevalent symptoms in both groups. Bajpai *et al.*'s cross-sectional study found that while fever and cough were common; the vaccinated group had more hemoptysis and taste alteration [11]. A prospective cohort analysis of 23,324 individuals in April 2021 found that vaccinated patients were 13% asymptomatic and 40% symptomatic, compared to 5% and 63% for the unvaccinated group [12]. In our study, hypertension (41.6%) was the most common co-morbidity, possibly due to SARS-CoV-2 binding on ACE-2 receptors, which raises angiotensin ll and stimulates RAAS, causing increased inflammation due to increased use of ACE inhibitors as anti-hypertensive medication. Diabetes (37.6%) was the second most common, followed by diabetes (37.6%). Surprisingly, 34.7% of patients had no comorbidities. Our investigation supports earlier findings that fully immunized patients had less comorbidity than unvaccinated patients [13]. In August 2022, 267 COVID-19-positive individuals were identified and comorbidities independently predicted mortality [14]. IL6 averaged 851.7 in non-vaccinated people and 161.0 in vaccinated people, suggesting T-cells release IL6 during COVID-19 infection.

Our study found that non-vaccinated individuals had higher levels of COVID markers like D-dimer, IL6, Serum Ferritin, HsCRP and LDH; however, only IL6 was significant. Studies reveal that vaccinated patients have considerably reduced C-reactive protein [15]. The vaccinated group had a lower average CT severity score (8.79) than the non-vaccinated group (10.5), while diabetics and hypertensives had higher CT scores, which may indicate a dysregulated immune response that increases pro-inflammatory activity and lung damage. Vaccinated people had a lower average CT severity score than unvaccinated people, which is consistent with Verma *et al.*'s retrospective cross-sectional investigation [16]. Vaccinated patients had higher oxygen saturation (91.53%) than non-vaccinated patients (84.92%) at admission. The need for oxygen was much reduced among vaccinated individuals (42.66%), compared to 26.92% of non-vaccinated patients. Compared to 8% and 12% for the vaccinated group, 19.23% and 15.38% of non-vaccinated patients needed CPAP and a ventilator. In our study of diabetics and hypertensives, the non-vaccinated group had higher average CT severity scores, longer hospital stays, and lower oxygen saturation at admission, the only significant finding. Abhilash *et al.* observed that two doses of vaccine significantly reduced oxygen use, hospitalization, NIV, and ICU admission in a cohort trial. One vaccine dose reduced oxygen need, hospitalization, and mortality on adjusted analysis [17]. Our study found that sepsis was more common (15.38%) in non-vaccinated patients than in vaccinated persons (1.3%). 13.3% of the vaccinated group and 44.4% of the non-vaccinated group died from the disease, indicating the vaccine's efficacy and impact on disease development. Vassallo *et al.* studied 126 patients in 2022 and found no difference in ICU transfer, mechanical ventilation, or death by vaccination status, but our study found a statistically significant difference [15]. A prospective observational study of 592 ICU patients found that most were unimmunized and had higher mortality rates [18]. A study of 232,268 COVID-19-positive patients, 4481 of whom were critically ill, found that the vaccine was 98% effective against death at 14 days or longer after the second dose and 77% at 14-21 days after the first dose. Most of the patients who died (64.2%) were unvaccinated. Our study also found 30.8% mortality in unvaccinated patients and 13.3% in vaccinated patients [19, 20]. The key strength of our study was assessing and comparing vaccinated and non-vaccinated patients in various ways to determine the COVID-19 vaccine's efficacy and illness progression. Historical COVID vaccination administration was recorded. We standardized all lab investigations and excluded outside investigations from the study. Since the second dose of the vaccine was available for everyone over 18, most individuals were immunized during the third wave from December 2021 to February 2022. Thus, this lets us research the virus's pathophysiology in the context of host immunity and provides raw data for vaccine efficacy in future pandemics.

## Conclusion:

COVID-19 vaccination significantly improves disease progression, reduces inflammatory marker levels (notably IL-6) and lowers the risk of severe complications, oxygen dependence and mortality. Vaccinated patients had better clinical outcomes, including higher oxygen saturation, lower CT severity scores and fewer ICU admissions. Thus, we show the importance of vaccination in mitigating COVID-19 severity, especially among individuals with comorbidities.

## Financial support and sponsorship:

Nil.
